# Chromatin regulators-related lncRNA signature predicting the prognosis of kidney renal clear cell carcinoma and its relationship with immune microenvironment: A study based on bioinformatics and experimental validation

**DOI:** 10.3389/fgene.2022.974726

**Published:** 2022-10-20

**Authors:** Xinyu Zhang, Xinyue Qin, Tiannan Yu, Kexin Wang, Yinhao Chen, Qianwei Xing

**Affiliations:** ^1^ Department of Urology, Affiliated Hospital of Nantong University, Medical School of Nantong University, Nantong, China; ^2^ Department of Laboratory Medicine, Affiliated Hospital of Nantong University, Nantong, Jiangsu, China; ^3^ Medical School of Nantong University, Nantong University, Nantong, Jiangsu, China

**Keywords:** prognosis, chromatin regulators-related lncRNA, signature, immune microenvironment, kidney renal clear cell carcinoma (KIRC)

## Abstract

**Background:** Kidney Renal Clear cell carcinoma (KIRC) is a major concern in the urinary system. A lot of researches were focused on Chromatin Regulators (CRs) in tumors. In this study, CRs-related *lncRNAs* (*CRlncRNAs*) were investigated for their potential impact on the prognosis of KIRC and the immune microenvironment.

**Methods:** The TCGA database was used to obtain transcriptome and related clinical information. CRs were obtained from previous studies, whereas *CRlncRNAs* were obtained by differential and correlation analysis. We screened the *lncRNAs* for the signature construction using regression analysis and LASSO regression analysis. The effectiveness of the signature was evaluated using the Kaplan-Meier (K-M) curve and Receiver Operating Characteristic curve (ROC). Additionally, we examined the associations between the signature and Tumor Microenvironment (TME), and the efficacy of drug therapy. Finally, we further verified whether these *lncRNAs* could affect the biological function of KIRC cells by functional experiments such as CCK8 and transwell assay.

**Results:** A signature consisting of 8 *CRlncRNAs* was constructed to predict the prognosis of KIRC. Quantitative Real-Time PCR verified the expression of 8 *lncRNAs* at the cell line and tissue level. The signature was found to be an independent prognostic indicator for KIRC in regression analysis. This signature was found to predict Overall Survival (OS) better for patients in the subgroups of age, gender, grade, stage, M, N0, and T. Furthermore, a significant correlation was found between riskScore and immune cell infiltration and immune checkpoint. Finally, we discovered several drugs with different IC50 values in different risk groups using drug sensitivity analysis. And functional experiments showed that Z97200.1 could affect the proliferation, migration and invasion of KIRC cells.

**Conclusion:** Overall, the signature comprised of these 8 *lncRNAs* were reliable prognostic biomarkers for KIRC. Moreover, the signature had significant potential for assessing the immunological landscape of tumors and providing individualized treatment.

## Introduction

Renal Cell Carcinoma (RCC) is among the ten most common forms of cancer globally (Siegel et al., 2018), second only to bladder cancer among urinary system tumors. There is no histological variety of RCC more common than Kidney Renal Clear cell Carcinoma (KIRC), accounting for 80%–90% of RCC. The KIRC is also the most common pathological variety causing death in renal cancer patients (Hsieh et al., 2017). There are more than 330,000 new cases of RCC globally and over 140,000 deaths each year and the incidence has continued to rise (Siegel et al., 2017). Advancements in medicine and the popularity of physical examinations have resulted in improvement in the medical level and an increase in the early diagnosis rate of KIRC. However, some patients have advanced KIRC at the time of diagnosis. Surgical treatment is preferred when KIRC is detected early. Because KIRC is not sensitive to radiotherapy and chemotherapy, targeted therapy is the main treatment for patients with advanced KIRC. According to statistical data, the prognosis of advanced KIRC is particularly poor, with a 5-year survival rate as low as 11.7% (Morrissey et al., 2015). Therefore, it is crucial to investigate the pathogenesis of KIRC, especially advanced KIRC. This type of research can also provide new insights into the clinical treatment of KIRC, and also provide potential molecular targets for the targeted therapy.

Noncoding RNAs have been extensively studied since the development of high-throughput technologies such as second-generation sequencing. Long noncoding RNAs (*LncRNAs*), a class of no protein genes coding potential, are initially assumed to be nonfunctional transcriptional byproducts. Studies on transcriptional activation, cell cycle regulation and epigenetic regulation (Miranda-Castro et al., 2019; Zhang et al., 2019) have been found for *lncRNAs* in the onset and development of disease. The occurrence and progression of tumors are influenced by more than a hundred *lncRNAs* with dysregulated expression, particularly urinary malignancies, as research advances. Liu et al. demonstrated that a signature composed of four *lncRNAs* can predict the prognosis of KIRC and that the signature could be used as a potential biomarker (Liu et al., 2020). Xia et al. validated a signature based on nine redox-related *lncRNAs* as a prognostic marker for KIRC (Qi-Dong et al., 2020). More studies have demonstrated that *lncRNAs* affect the prognosis of KIRC, implying that we may be able to develop more accurate biomarkers of KIRC based on *lncRNAs*.

Chromatin Regulators (CRs) are a class of proteins mainly involved in the fine regulation of chromatin structure (Gonzalez-Perez et al., 2013). The CRs are mainly composed of DNA methylators, histone modifiers, and chromatin remodelers. To participate in the biological process of the tumor, CRs can promote epigenetic changes. Polybromo-1 (*PBRM1*), a chromatin regulator, has been identified as the most mutated gene in KIRC and a potential target for KIRC therapy (Aili et al., 2021). A recent study recommended 11 CRs as a biomarker for bladder cancer (Zhu et al., 2022). However, no study has been conducted to investigate the role of CRs-related *lncRNAs (CRlncRNAs)* in KIRC. In the study, we employed bioinformatics to construct a signature of *CRlncRNAs* and analyzed if it could be used for KIRC.

## Method and materials

### Collection and processing of data

Data on all KIRC transcriptomes, their clinical characteristics and mutation data were obtained from The Cancer Genome Atlas (TCGA) database (https://www.cancer.gov/), excluding samples with missing clinical information. Mutation data was downloaded to analyze the association between the signature and tumor mutational burden (TMB). From the previous study, we obtained 870 CRs (Lu et al., 2018). Differential analysis of these regulators was done using |logFC| > 1 and False Discovery Rate (FDR) < 0.05 as screening conditions, to acquire Differentially Expressed CRs (DECRs) in KIRC. In addition, *CRlncRNAs* were selected by Pearson correlation analysis, with correlation coefficients higher than 0.8 and *p*-values below 0.05. Finally, differential analysis was used to identify Differentially Expressed *CRlncRNAs* (DECRLs), with the same conditions as before.

### Construction of a prognostic signature based on chromatin regulators-related lncRNAs

To generate training and testing sets, a 7:3 split of the entire TCGA dataset was performed. We used the training set for signature construction. A testing set was used to demonstrate the value of the signature and the entire set. The training set was first subjected to univariate regression analysis to identify DECRLs that affect the prognosis of KIRC. In addition, Least Absolute Selection Operator (LASSO) regression analysis was employed to avoid overfitting. These *lncRNAs* were used to construct a prognostic signature for calculating risk scores for KIRC patients. The formula for calculating risk scores was: Risk score = $\overset{n}{\underset{i=1}{\sum }}expi*\beta i$ , where β represented the coefficient value of *lncRNAs* and exp denotes the expression level.

### Validation of the prognostic signature

The prognostic model was further validated using a testing and the entire set. Patients were classified into high- and low-risk groups based on their median risk score. Based on the Kaplan-Meier (K-M) curve, we compared survival difference of different groups of patients. We also calculated Area Under the Curve (AUC) and assessed the accuracy of the signature in predicting KIRC prognosis using the Receiver Operating characteristic Curve (ROC). The differential expressions of eight *lncRNAs* were compared between different groups using heatmaps.

### Validation of prognostic signature as an independent prognostic factor

Based on logistic regression analysis, the correlations between clinicopathological factors and riskScore were calculated and presented in the form of a heatmap. The prognostic value of the riskScore was investigated using univariate/multivariate regression analysis. The ROC was subjected to compare the accuracy of signature and several clinical characteristics in the prediction of KIRC prognosis.

### Construction and evaluation of nomogram

As per the previous methods (Iasonos et al., 2008), a nomogram was developed based on 8 clinical characteristics and riskScore to assess Overall survival (OS) in patients with KIRC. Additionally, calibration curves were used to assess the nomogram for OS.

### Enrichment analysis and gene set variation analysis (GSVA)

Annotating differentially expressed genes involved the use of Gene Ontology (GO) analysis, which was comprised of three processes, including biological process, molecular function and cellular components. The Kyoto Encyclopedia of Genes and Genomes (KEGG) pathway analysis was applied in the investigation of the relevant pathways to further analyze the mechanisms associated with prognostic models in KIRC. The GSVA was an algorithm that can calculate the variance scores for specific sets of genes in each sample, without the need for prior variance analysis between samples. GSVA was performed on the entire as per the previous methods (Hänzelmann et al., 2013), and Person correlation analysis was used to assess the correlation of GSVA scores with prognostic models and the 8 *lncRNAs*.

### Assessment of tumor microenvironment and immune cell infiltration

We used the ESTIMATE algorithm to calculate ImmunityScore, StromalScore and ESTIMATEScore for different risk groups for improving understanding of the association between immunity and riskScore. The CIBERSORT (Newman et al., 2015), CIBERSORT-ABS, TIMER (Li et al., 2017), xCELL (Aran et al., 2017), MCPcounter (Dienstmann et al., 2019), QUANTISEQ and EPIC (Racle and Gfeller, 2020) were used to assess immune cell infiltration in samples of the entire TCGA set and to establish the association between riskScore and immune cell infiltration. In this study, we investigate the level of tumor-infiltrating immune cells and assessed their immunological activities using ssGSEA.

### Prediction of drug sensitivity

Immune checkpoint expression in different groups was examined using the Wilcoxon signed-rank test. We further evaluated the usefulness of prognostic signature in predicting the effect of drug treatment for KIRC. For the purpose of determining sensitivity to drugs, the half-maximal inhibitory concentration (IC50) of different samples was calculated using the pRRophetic package (Geeleher et al., 2014). A lower IC50 value was indicative of higher drug sensitivity.

### Validation of the expression of *lncRNAs* in KIRC based on quantitative real-time PCR (qRT-PCR)

Verification of gene expression was conducted at tissue level and cellular level. Validation at the cellular level was accomplished using normal renal tubular epithelial cells (HK-2) and renal tumor cells (ACHN, 769-P, 786-O). In addition, we collected KIRC and adjacent normal tissue samples from nine pairs of KIRC patients who underwent surgery at Nantong University Hospital for tissue-level validation. TRIzol reagent was used to extract RNA as per the instructions of the vendor. The reverse transcription kit (Vazyme, Nanjing, China) was then used to convert RNA to cDNA. The SYBR Green was used for qRT-PCR. The primer sequences for *lncRNAs* were presented in Table 1.

**TABLE 1:** Primer sequence of lncRNAs.

Order	Primer	5′ to 3′
1	LINC00551_F	TGC​CTA​TAG​GTG​CCA​AGA​CC
2	LINC00551_R	TCT​CCA​CCT​GAC​ATC​CCT​TC
3	AL031722.1_F	CTC​AAG​CGA​TCG​ACC​AGT​CT
4	AL031722.1_R	CTC​CTG​GGT​TCA​AGC​AAT​TC
5	AC093001.1_F	GCG​GAA​GCT​TTG​TTC​TTT​TG
6	AC093001.1_R	TCG​CGG​TGT​TAC​AGC​TCA​TA
7	NDUFB2-AS1_F	TAA​TGC​CTG​CAA​GTG​GAC​AG
8	NDUFB2-AS1_R	GCT​TGG​CCA​CTT​CCT​TAA​CA
9	LINC00894_F	TGA​GCT​GCT​CCT​CAC​TCT​CA
10	LINC00894_R	ATC​CGA​CCA​CAG​ATC​AGA​CC
11	Z97200.1_F	ATC​AGG​GAA​GAG​GGG​AGT​GT
12	Z97200.1_R	TTC​ATC​CCT​GAG​TCC​CTT​TG
13	AC006160.1_F	GAA​TTC​TGG​TCG​GAG​ATG​GA
14	AC006160.1_R	CCC​TGA​TCA​TGA​CAC​TGC​AC
15	AC092422.1_F	GCT​GAC​TCG​TCC​CTT​TTC​TG
16	AC092422.1_R	TCC​TCC​AGA​TGA​GCA​GGA​CT

The relative expression of LncRNA was evaluated using the 2^−ΔΔCt^ method and plotted by Graphpad Prism 8. The Institutional Research Ethical Committee of Nantong University Hospital Institutional Research Ethics Committee granted research ethics approval for this study.

### Cell culture and cell transfection

ACHN and 769-P cells (Shanghai Institute for Biological Sciences) were cultured in a constant temperature incubator at 37°C with a CO_2_ volume fraction of 5%. Cells were spread into 6-well plates and transfected the next day. The interference plasmids were obtained from GenePharma (Shanghai, China). And each 6-well plate was replaced with 2 ml complete medium +3.75 µl LipofectamineTM 3000 + 2500 ng negative control or sh-RNA when the cell density reached about 70%. After 12 h, the complete medium containing serum and antibiotics was replaced with 2 ml. 48 h after transfection, the cells were collected and used for subsequent experiments.

### Cell function experiments

After cells were transfected for 48 h, cells were spread evenly in 96-well plates, and 5 parallel replicate wells were set up for each group of fragments. After waiting for cell apposition according to the cell characteristics, 10 μl CCK-8 reagent was added separately for 5 consecutive days, and the absorbance at 450 nm and 630 nm was detected by enzyme marker at the same time every day. The 24-well plates were infiltrated using 200 μl of culture medium. After that, transwell chambers were added, and the stromal gel and medium were configured according to 1:6 in advance in the invasion experiment and put into the chambers. The configured cell suspensions were added to the chambers and fixed after 48–72 h using 4% paraformaldehyde, stained with crystal violet, rinsed with PBS and photographed.

## Results

### Identification of differentially expressed CRlncRNAs (DECRLs)

**FIGURE 1:**
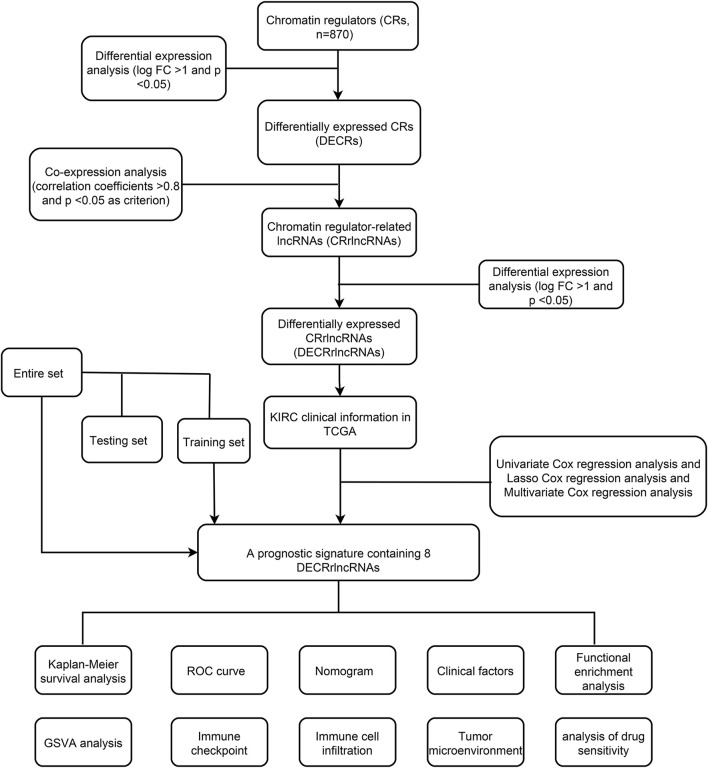
Workflow of this study.

**FIGURE 2:**
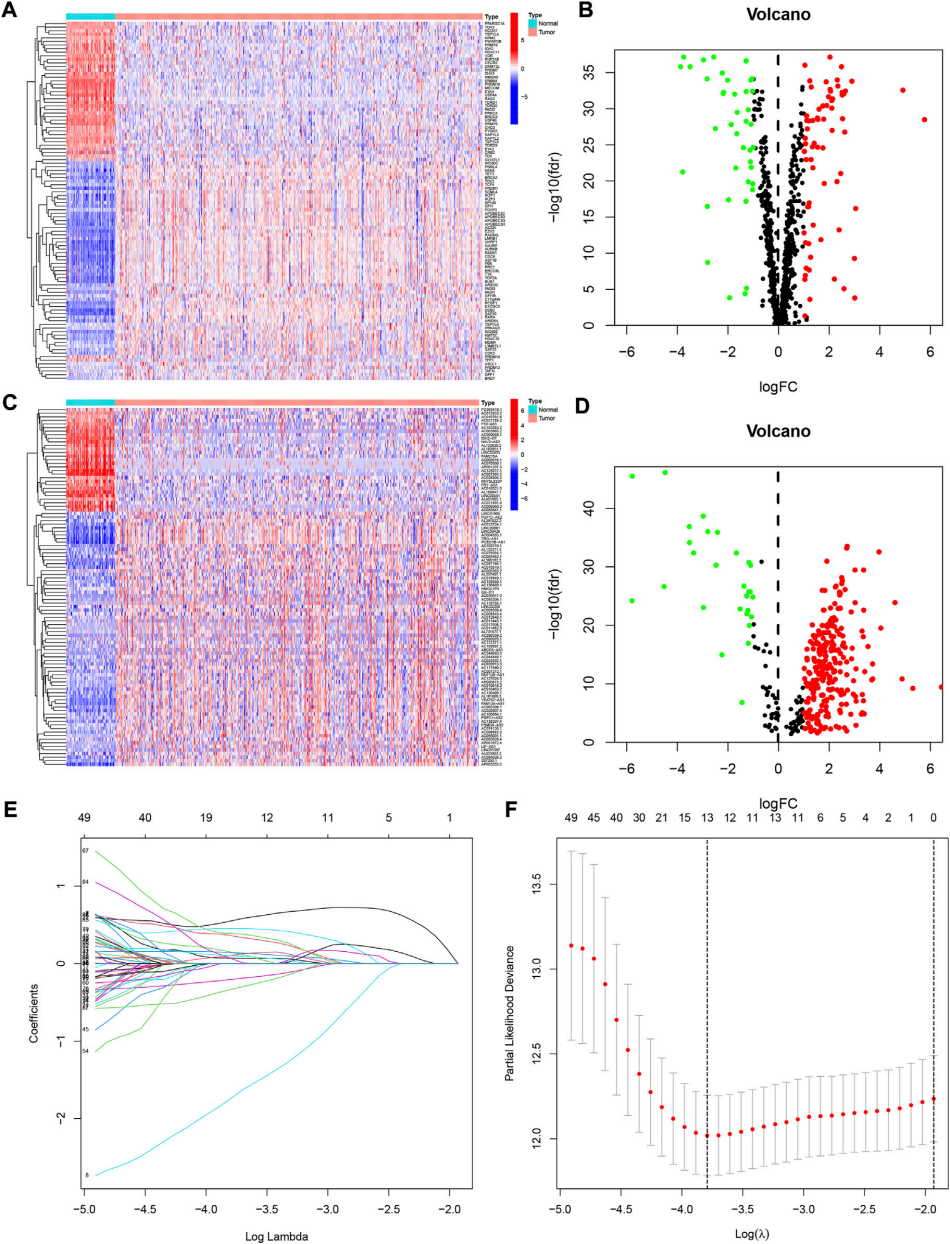
The differentially expressed chromatin regulator-related genes identified in KIRC. **(A)** Heatmap of these differently expressed chromatin regulators. **(B)** Volcano plot of these differently expressed chromatin regulators. **(C)** Heatmap of these differently expressed chromatin regulators-related lncRNAs. **(D)** Volcano plot of these differently expressed chromatin regulators-related lncRNAs (CRlncRNAs). **(E,F)** Lasso regression analysis of CRlncRNAs and calculation of the minimum criteria.

Figure 1 showed the flow chart of the whole paper. First, we retrieved 870 CRs from a previous study and downloaded the entire transcriptome data of KIRC including 539 tumors and 72 paracancerous tissue samples in the TCGA database. The 870 CRs were screened by differential analysis in the first step. Figures 2A,B shows DECRs as heatmaps and volcano maps. We then obtained 287 DECRLs, including 258 up-regulated *lncRNAs* and 29 down-regulated *lncRNAs* (Figures 2C,D).

### Construction and validation of a prognostic signature for DECRLs

**FIGURE 3:**
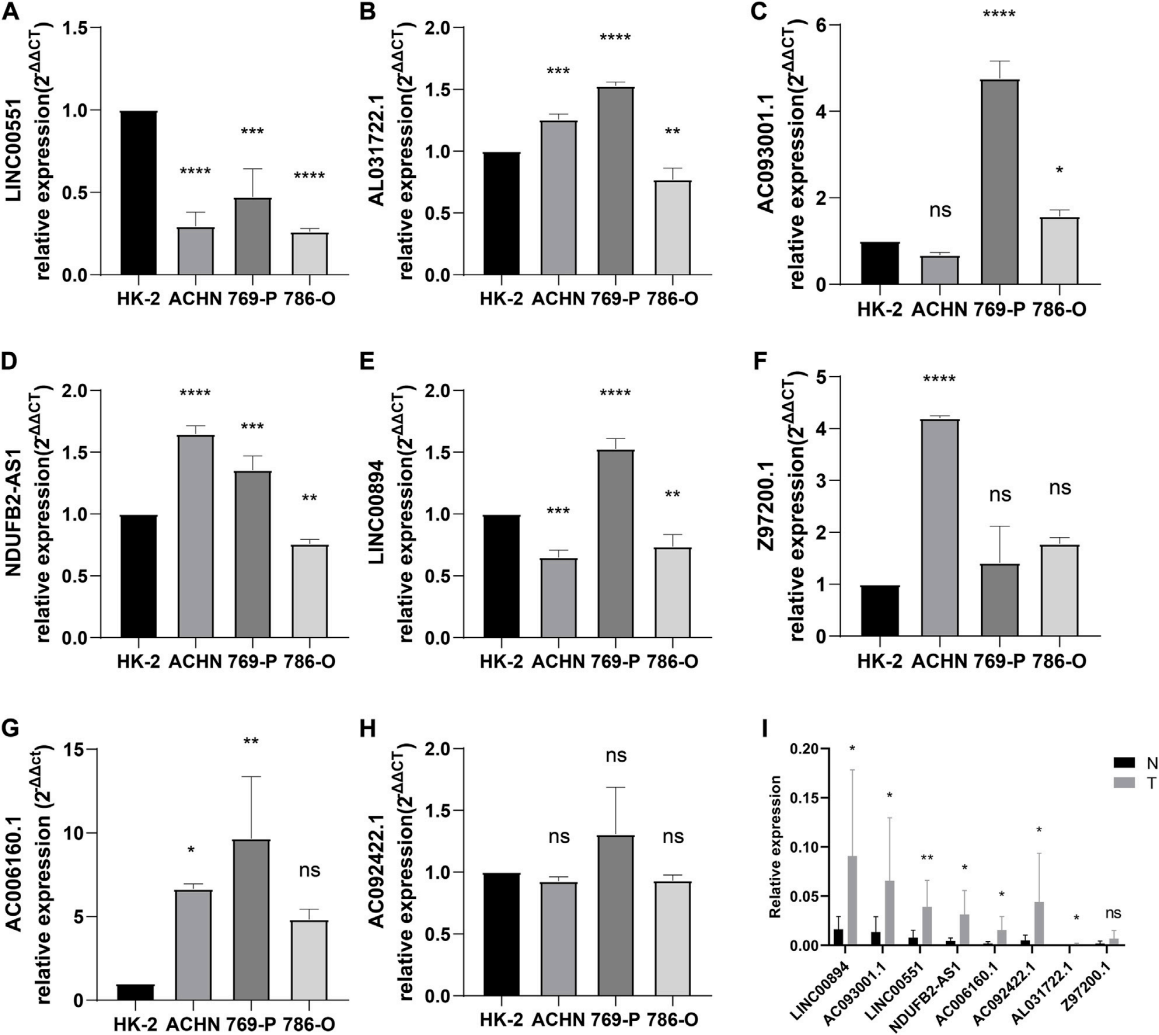
Verification of gene expression. **(A)** LINC00551, **(B)** AL031722.1, **(C)** AC093001.1, **(D)** NDUFB2-AS1, **(E)** LINC00894, **(F)** Z97200.1, **(G)** AC006160.1, and **(H)** AC092422.1 expression in normal and kidney cancer cell lines; **(I)** eight lncRNAs expression in normal kidney tissue and KIRC tissue. *p < 0.05; **p < 0.01; ***p < 0.001; ****p < 0.0001.

We developed a prognostic signature for patients with KIRC using TCGA training set. First, univariate regression analysis was conducted to obtain 99 lncRNAs associated with KIRC prognosis (Supplementary Table S1). Then, LASSO and multivariate regression analysis were employed to identify 8 *lncRNAs* (LINC00551, AL031722.1, AC093001.1, NDUFB2-AS1, LINC00894, Z97200.1, AC006160.1, and AC092422.1) that were involved in the development of risk model (Figures 2E,F). Using the training set, we performed K-M survival analysis on 8 *lncRNAs* (Supplementary Figure S1). Patients with high expression of AC006160.1, AC093001.1, LINC00894, NDUFB2-AS1, and Z97200.1 were predicted to have a poor prognosis, whereas patients with high expression of AC092422.1, AL031722.1, and LINC00551 were expected to have a better prognosis. Moreover, the risk score was computed as: risk score = (−3.2301 * LINC00551 expression) + (−0.4501 * AL031722.1 expression) + (0.2268 * AC093001.1 expression) + (0.7924 * NDUFB2-AS1 expression) + (0.5056 * LINC00894 expression) + (0.5577 *Z97200.1 expression) + (0.9458 * AC006160.1 expression) + (−0.9352 * AC092422.1 expression). We validated the expression of these eight *lncRNAs* using PCR at the tissue level and cellular level (Figure 3). AC092422.1 expression in the three kidney cancer cells was not statistically significant. The rest of the genes were significantly different in the expression in the kidney cancer cell lines. For instance, the expression of LINC00551 decreased in ACHN, 769-P and 786-O cells. Moreover, tissue-level expression results revealed that seven genes were highly expressed in KIRC, which was slightly different from the cellular level expression results.

### Survival results analysis and model validation

**FIGURE 4:**
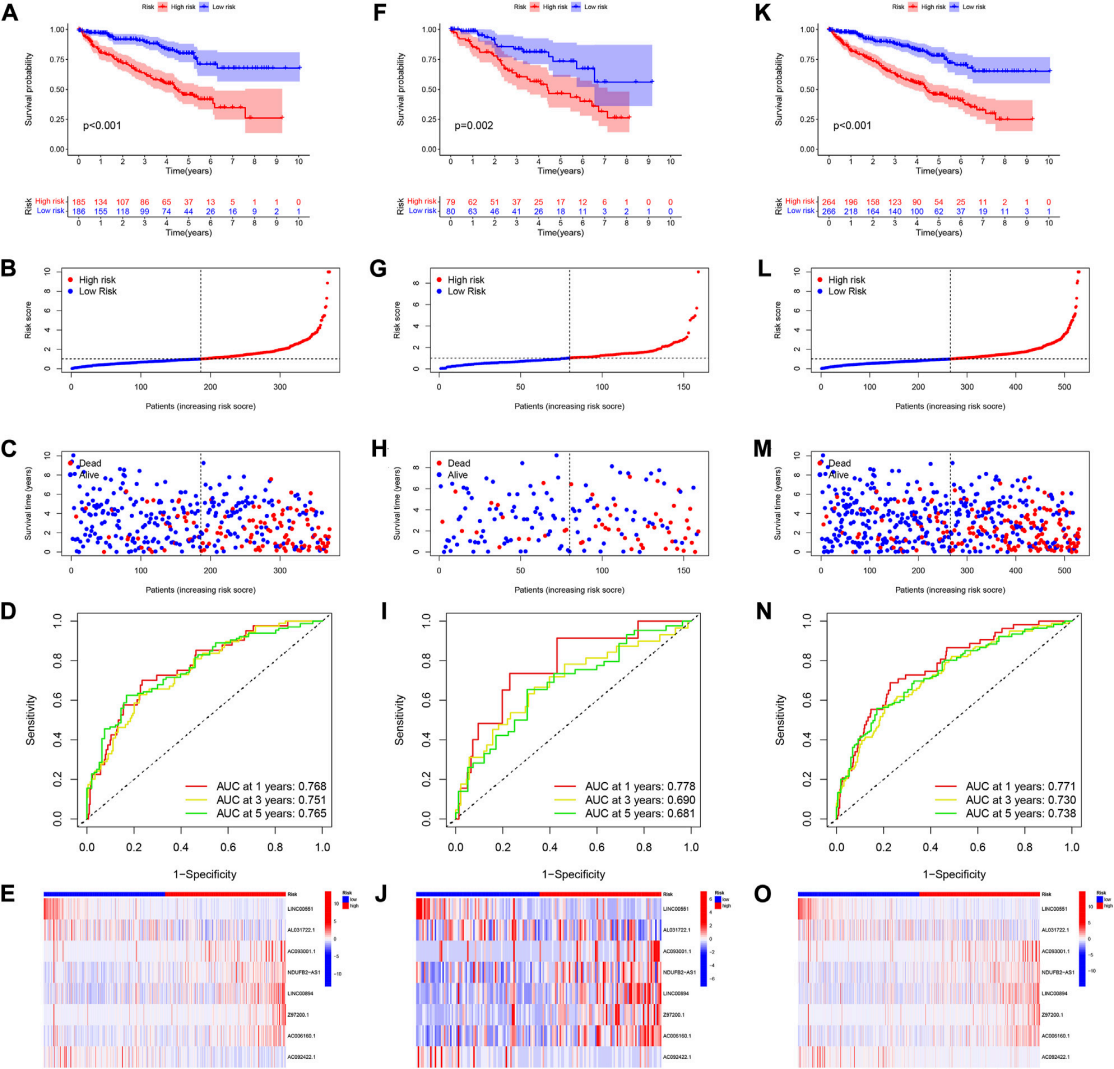
Verification of 8 CRlncRNAs signature. **(A–E)** KM survival, risk score, 1-, 3-, and 5-year ROC and heatmaps of according to CRlncRNAs groups in TCGA training set. **(F–J)** KM survival, risk score, 1-, 3-, and 5-year ROC and heatmaps of according to CRlncRNAs groups in TCGA testing set. **(K–O)** KM survival, risk score, 1-, 3-, and 5-year ROC and heatmaps of according to CRlncRNAs groups in the entire TCGA set.

The patients were divided into two risk groups including high-risk and low-risk. Patients in the high-risk category had poorer survival (Figure 4A). Figures 4B,C showed the survival status and distribution of patients, with higher scores accounting for more deaths. The ROC was also used to validate the signature for prognostic prediction. The signature had AUC values of 0.768, 0.751, and 0.765 for 1-, 3-, and 5-year, indicating that it had good predictive efficacy (Figure 4D). The expressions of these 8 *lncRNAs* in high- and low-risk groups were displayed in Figure 4E. We used both the testing and entire set to verify the reliability of the signature. The results of the testing set implied that high-risk patients had a worse prognosis (Figures 4F–H). Additionally, the results of the ROC analysis indicated moderate accuracy (Figure 4I). Figure 4J depicted a heatmap of 8 *lncRNAs* differentially expressed in high- and low-risk groups. The validation results for the entire testing set exhibited the same trend (Figure 4K−O).

### The signature was an independent prognostic factor for KIRC

**FIGURE 5:**
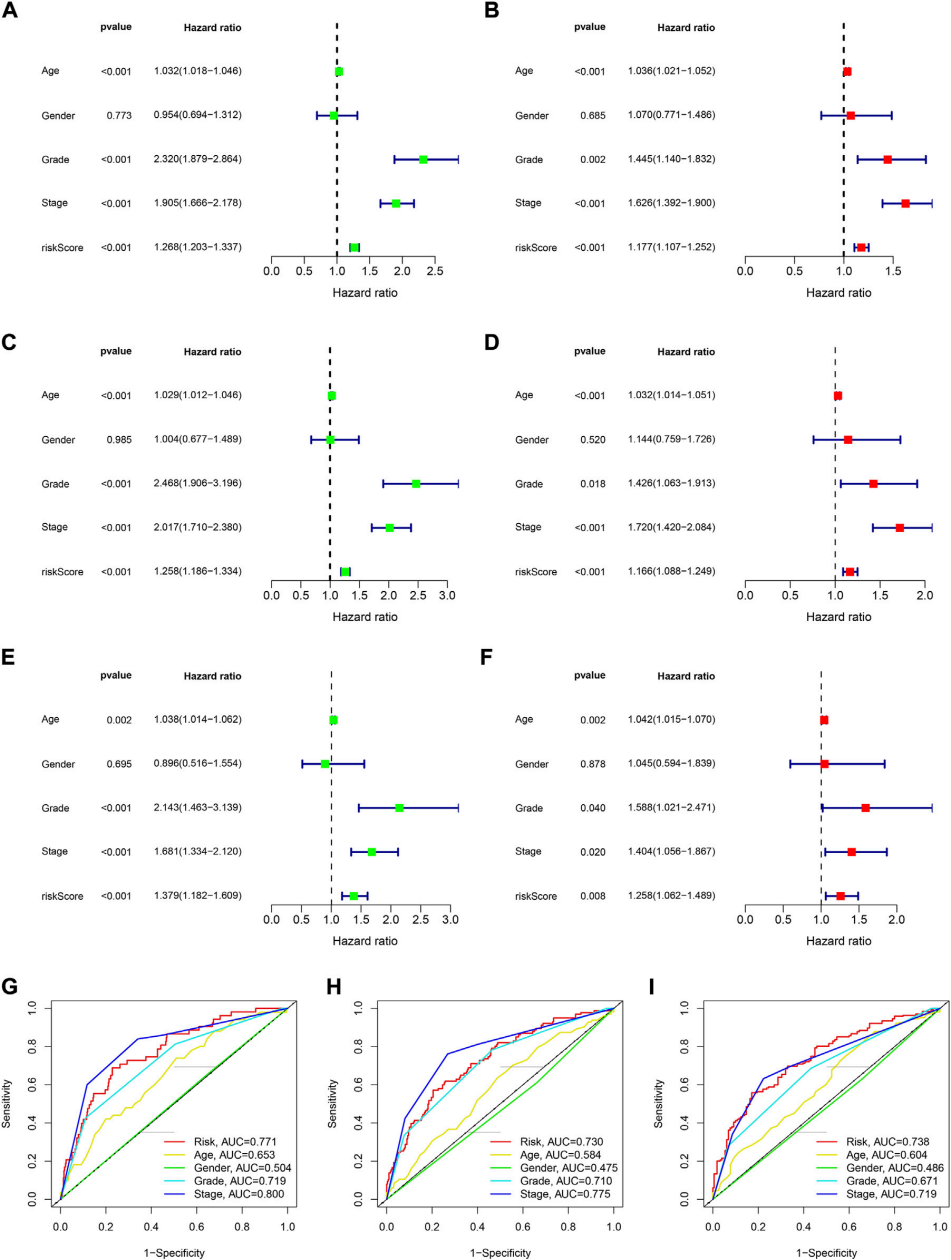
The assessment of independent prognostic factor. **(A,B)** Univariate and multivariate Cox regression analysis of the entire dataset (TCGA). **(C,D)** Univariate and multivariate Cox regression analysis of the training dataset. **(E,F)** Univariate and multivariate Cox regression analysis of the testing dataset. **(G–I)** 1-, 3- and 5-year ROC curves of riskScore and other clinicopathologic characteristics.

We used univariable and multivariable Cox analysis on three datasets to investigate the prognostic significance of the signature in KIRC. In the entire set univariable/multivariable Cox analysis revealed that riskScore could serve as an independent prognostic factor of KIRC (univariable Cox analysis: HR = 1.268 and *p* < 0.001, multivariable Cox analysis: HR = 1.177 and *p* < 0.001; Figures 5A,B). Meanwhile, the results of univariable/multivariable Cox analysis in the training and testing set all suggested that riskScore could independently affect the prognosis of KIRC (univariable Cox analysis: HR = 1.258 and *p* < 0.001, multivariable Cox analysis: HR = 1.166 and *p* < 0.001 (training set); univariable Cox analysis: HR = 1.379 and *p* < 0.001, multivariable Cox analysis: HR = 1.258 and *p* = 0.008 (testing set) (Figures 5C–F). All of the results indicated that the signature could affect the prognosis of KIRC independently (Supplementary Table S2).

### The relationship between prognostic signature and clinical characteristics

**FIGURE 6:**
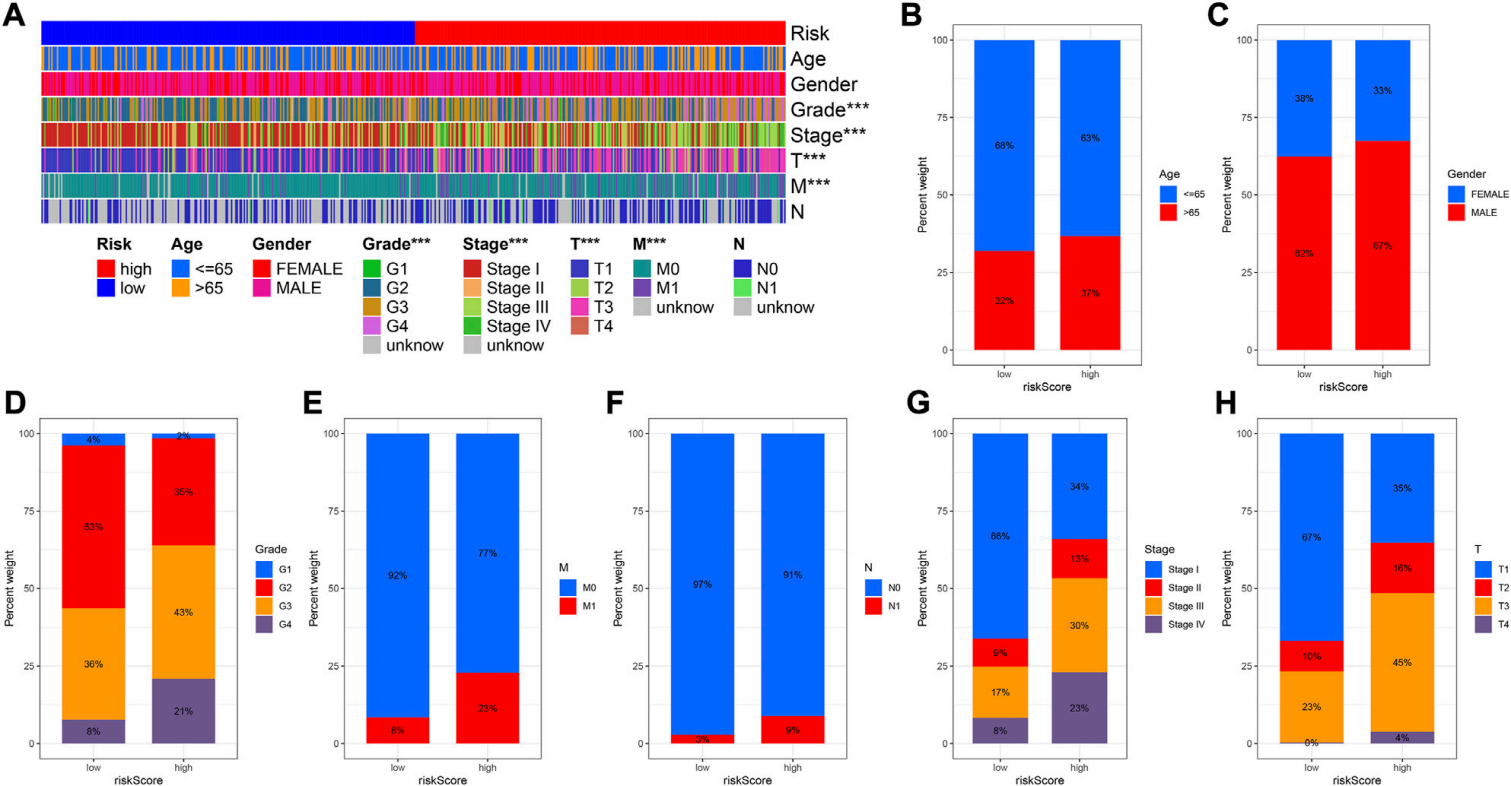
Correlation between risk score and clinicopathological factors. **(A)** Heatmap for CRlncRNAs prognostic signature and clinicopathological factors. **(B–H)** Proportion and distribution of patients with different clinical traits in high- and low-risk groups.

**FIGURE 7:**
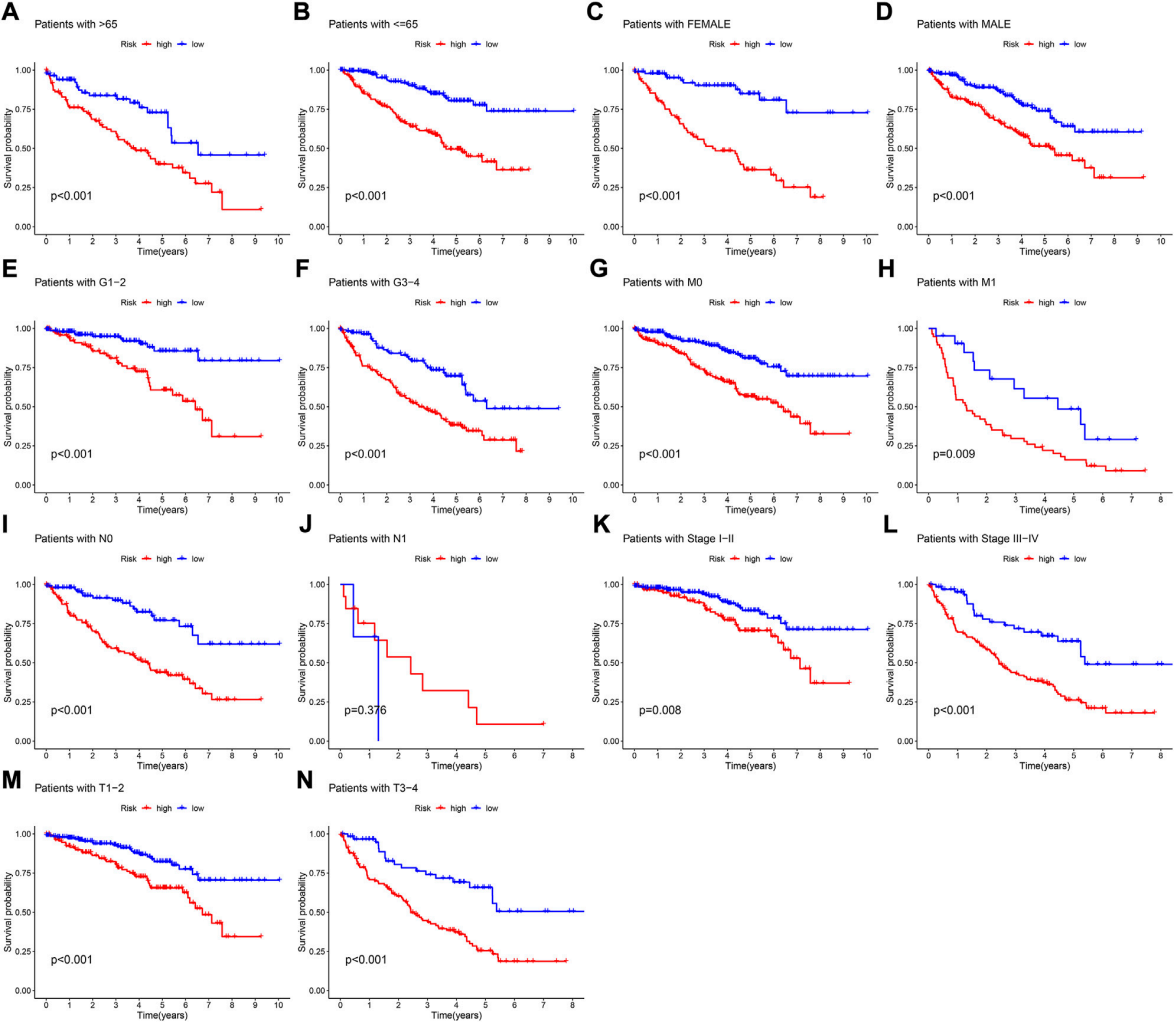
K-M survival curves of patients with different clinical traits. **(A)** Age >65 ranked by risk score for OS. **(B)** Age< = 65 ranked by risk score for OS. **(C)** Female ranked by risk score for OS. **(D)** Male ranked by risk score for OS. **(E)** Grade1-2 ranked by risk score for OS. **(F)** Grade3-4 ranked by risk score for OS. **(G)** M0 ranked by risk score for OS. **(H)** M1 ranked by risk score for OS. **(I)** N0 ranked by risk score for OS. **(J)** N1 ranked by risk score for OS. **(K)** Stage I-II ranked by risk score for OS. **(L)** Stage III-IV ranked by risk score for OS. **(M)** T1-2 ranked by risk score for OS. **(N)** T3-4 ranked by risk score for OS.

The results of 1- and 3-year ROC curves showed that riskScore and stage had higher sensitivity and specificity in predicting OS of patients with KIRC than other factors. Furthermore, the results of 5-year ROC curve showed that riskScore had the highest sensitivity and specificity (Figures 5G–I). The heatmap depicted the relationships between riskScore and clinical variables, where riskScore differed significantly in grade, stage, T, and M (Figure 6A). Column charts were used to show the proportion and distribution of patients with various clinical traits in the high and low-risk groups (Figures 6B–H). Patients in the high and low-risk groups had the similar age, sex, and N percentages (Figures 6B,C,F). On the contrary, there were differences in the composition of grade, stage, T, and M in the high and low-risk groups of patients. We further investigated the effect of prognostic signature in different clinical subgroups on the prognosis of patients with KIRC. Except for N1, all the signature predicted a better prognosis for KIRC patients with different clinical traits (Figure 7). Survival did not differ significantly among N1 patients with different risk scores.

### Construction of nomogram, GO, KEGG, and GSVA

**FIGURE 8:**
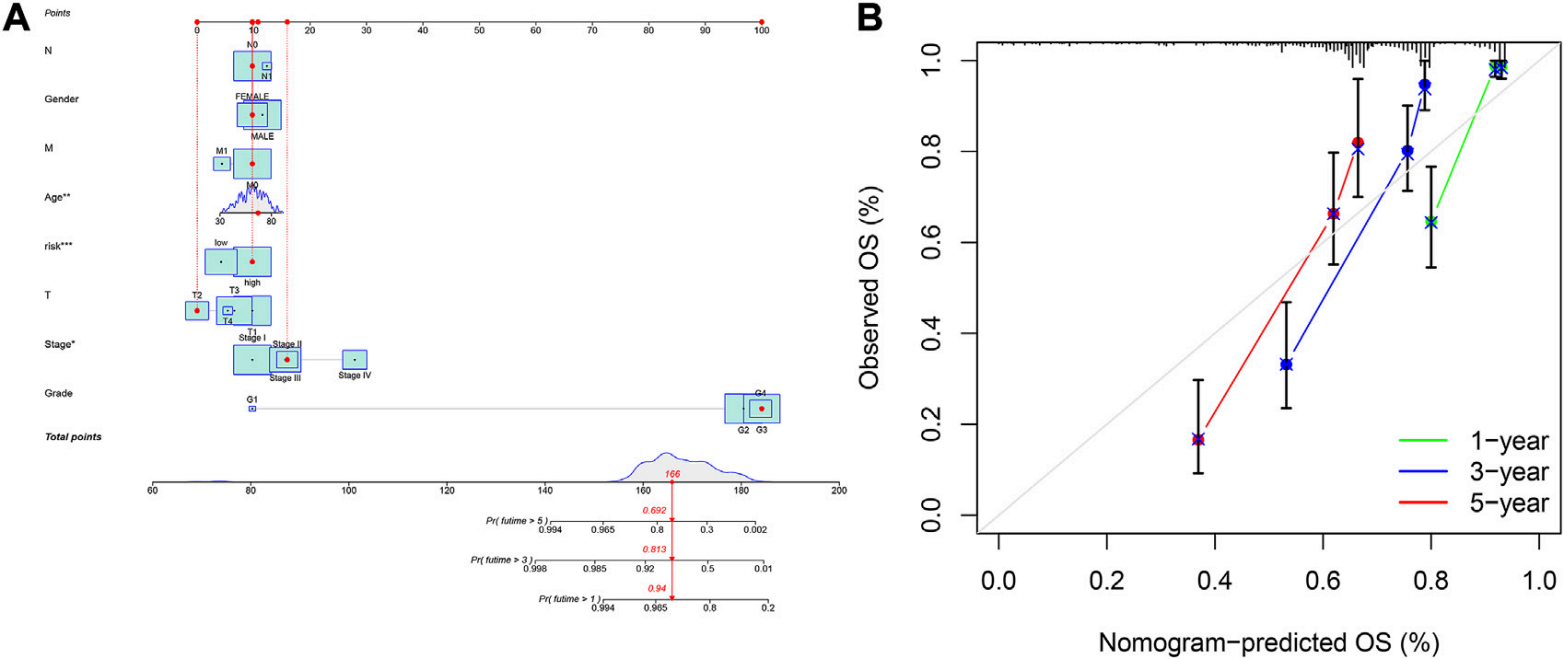
Construction of nomogram based on the signature and clinicopathological factors. **(A)** nomogram for predicting 1-, 3-, and 5-year OS. **(B)** The calibration plots for predicting 1-, 3-, and 5-year OS.

**FIGURE 9:**
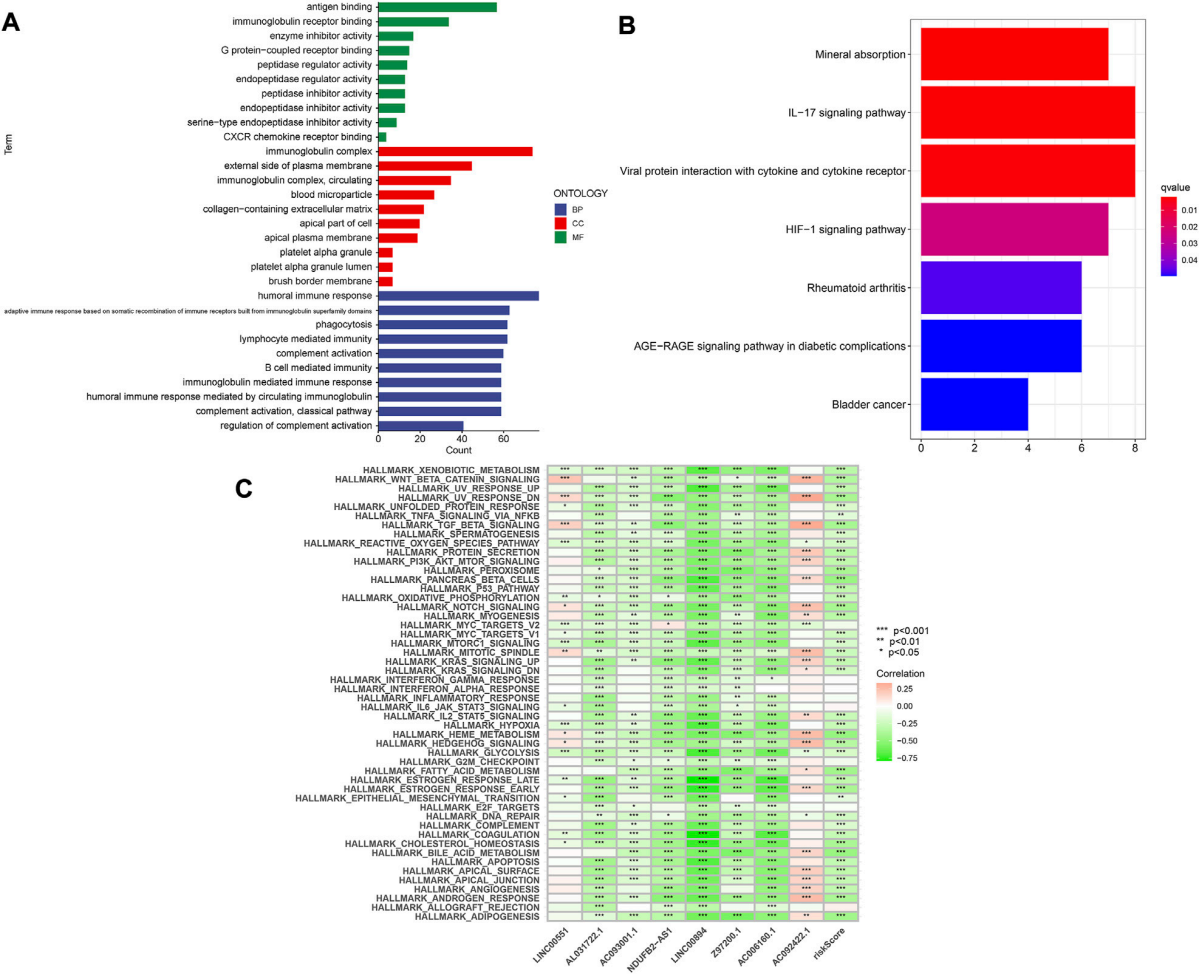
Enrichment analysis of differentially expressed genes. **(A)** GO analysis. **(B)** KEGG analysis. **(C)** GSVA analysis.

A nomogram containing clinical factors and riskScore can be used for the prognosis of KIRC patients (Figure 8A). The OS of KIRC patients can be predicted by calculating the total score. The calibration curve results suggested that the nomogram plot had a good predictive ability (Figure 8B). We conducted differential analyses, GO and KEGG analyses on high and low-risk groups. These genes were mainly involved in antigen binding and immunoglobulin receptor binding in terms of molecular function. Cellular component analysis revealed that these genes were enriched in the immunoglobulin complex, the external side of the plasma membrane, among others. The biological process results revealed that they were primarily related to humoral immune response and phagocytosis (Figure 9A). According to KEGG analysis, they were involved in mineral absorption, IL−17 signaling pathway, viral protein interaction with cytokine and cytokine receiver, HIF−1 signaling pathway and so on (Figure 9B). We performed GSVA and correlation analysis to further explore the pathways associated with risk score. The GSVA results showed that signaling pathways such as UV_RESPONSE_DN, TGF_BETA_SIGNALING, and MITOTIC_SPINDLE were significantly associated with 8 *lncRNAs* (Figure 9C). Many pathways including ADIPOGENESIS, ANDROGEN_RESPONSE, and ANGIOGENESIS were significantly negatively associated with risk score. This suggested that there could be an association between the pathways and the development of KIRC.

### Correlation between prognostic signature and tumor microenvironment and immune cells infiltration

**FIGURE 10:**
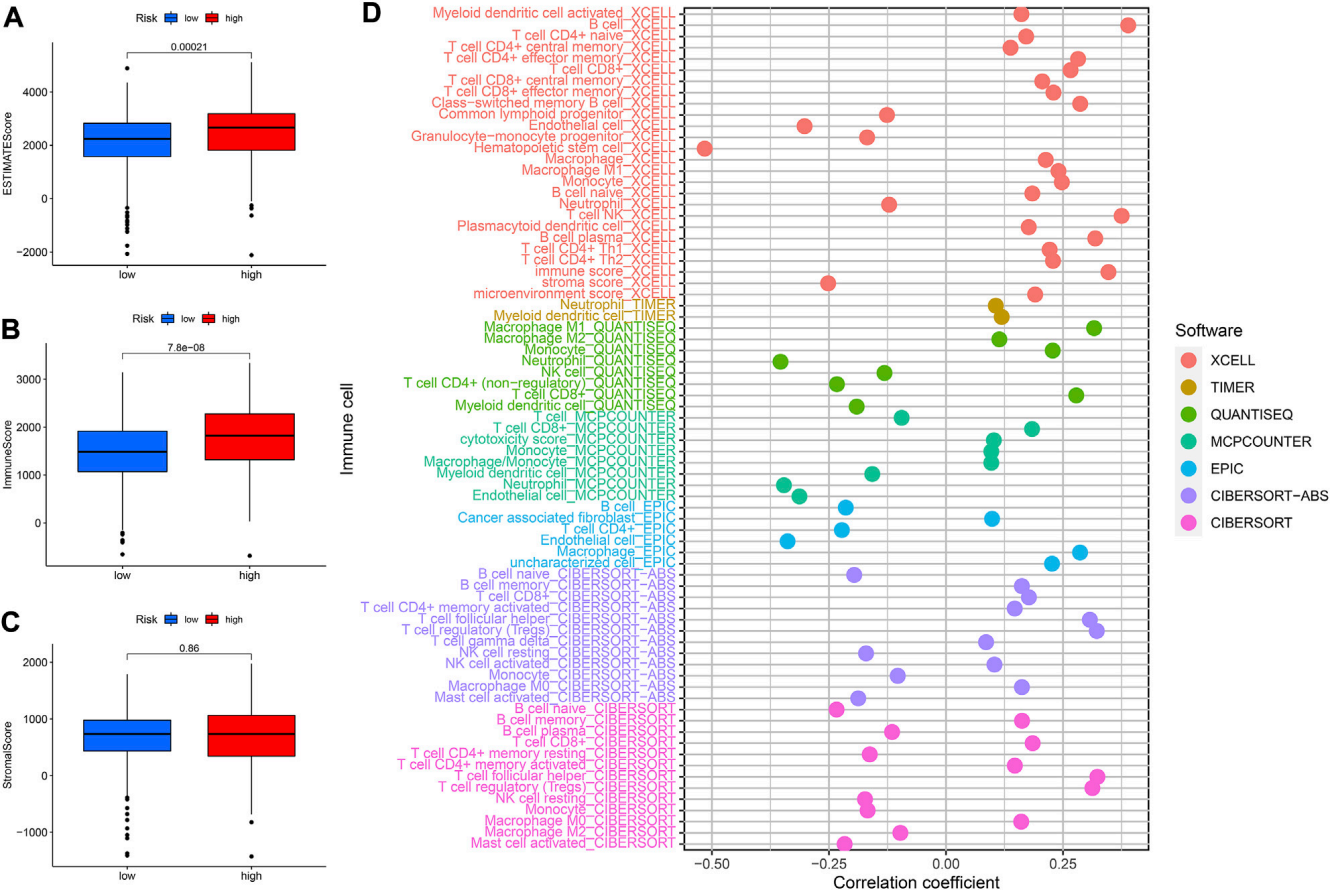
Analysis of immune landscape. **(A–C)** The relationship between prognostic signature and TME. **(D)** The relationship between immune cells and risk score was explored by correlation analysis.

It was well known that TMB was an important marker for tumor treatment. We further explored the relationships between TMB and risk scores as well as OS. We found that the higher the risk score the higher the TMB (Supplementary Figure S3A). Besides, patients in the high-TMB high-risk group had the worst prognosis, while those in the low-TMB low-risk group had the best prognosis compared to the other two groups (Supplementary Figure S3B). The stromal score and immune score were assessed in different risk groups to further examine the TME. The high-risk group had higher ESTIMATEScore and ImmuneScore, but there were no significant differences in StromalScore between the two groups (Figures 10A–C). The XCELL algorithm results indicated that the riskScore was significantly positively related to B cell, CD4^+^effector memory T cell, CD8^+^T cell, whereas it was negatively correlated with endothelial cell and so on (Figure 10D). The QUANTISEQ algorithm results revealed that riskScore was significantly positively associated with M1 Macrophage, among others, while negatively associated with neutrophils. The results of the EPIC algorithm showed that riskScore was significantly positively related to Macrophage while negatively correlated with endothelial cells. Moreover, we investigated the relationship between these 8 *lncRNAs* and immune cell infiltration (Supplementary Figure S2). The results found that LINC00894 was positively related to CD4^+^ central memory T cells (R = 0.41, *p* < 2.2e^−16^, Supplementary Figure S2A). NDUFB2−AS1 negatively correlated with endothelial cells (Supplementary Figures S2B,C). The Z97200.1 was positively correlated with NK T cell (R = 0.42, *p* < 2.2e^−16^).

### Associations between prognostic signature and immune checkpoint and immune functions

**FIGURE 11:**
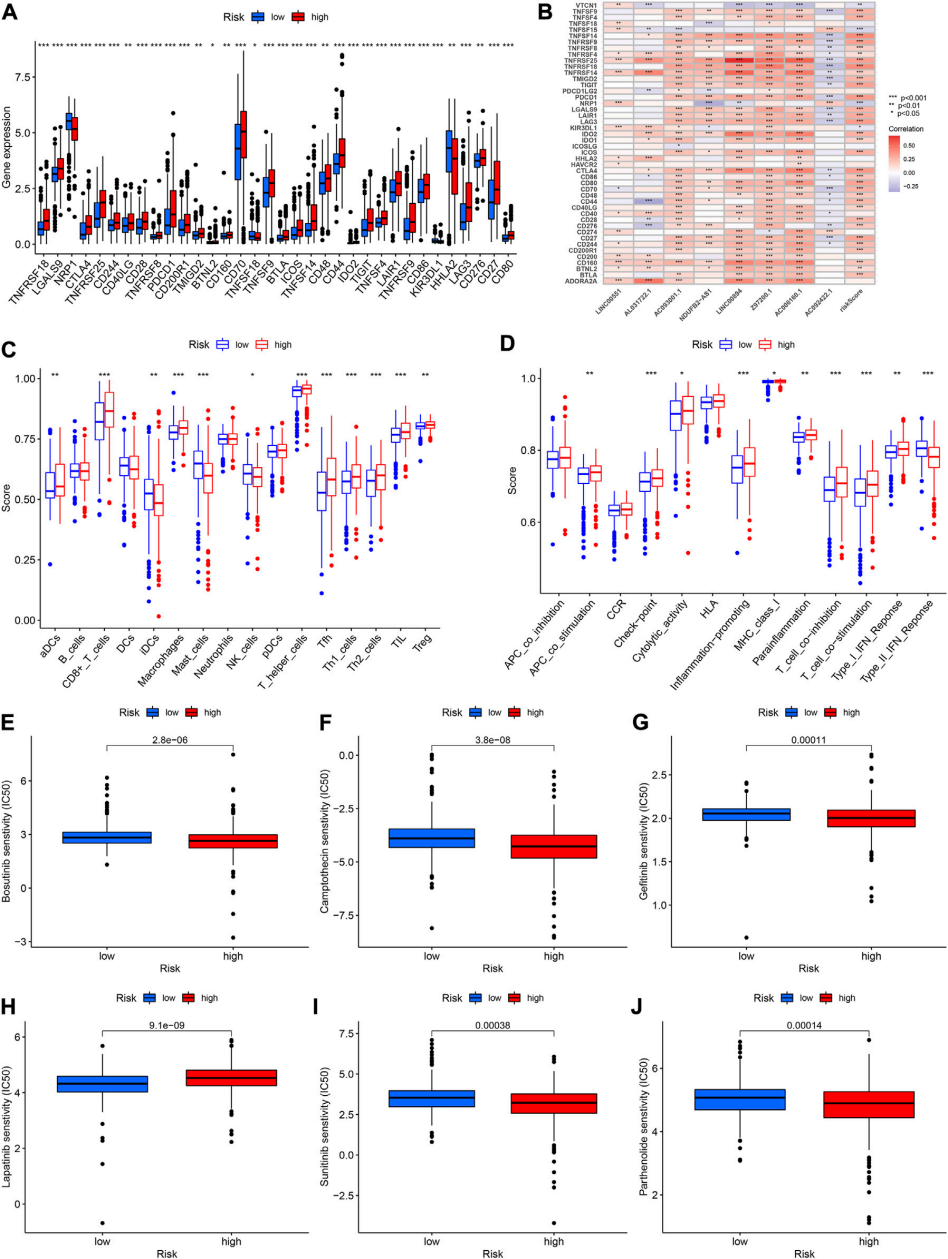
**(A)** Expression of immune checkpoints in high- and low-risk groups. **(B)** Relationships between immune checkpoint and risk score and lncRNAs. **(C,D)** Comparison of the scores of immune cells and immune functions between high- and low-risk groups. **(E–J)** The abilities of the risk model to predict drug sensitivity.

We performed correlation analysis as well as explored the expression of immune checkpoints in different risk score groups since the study of immune checkpoints can be of great help in immunotherapy. The high-risk group had higher expression of CTLA4, LAG3, PDCD1 and other immune checkpoints than the low-risk group (Figure 11A). The CTLA4, PDCD1, and TNFSF14 were significantly and positively correlated with riskScore (Figure 11B). In addition, CD44 was significantly negatively associated with AL031722.1 while TNFRSF25 was significantly positively associated with LINC00894 (Figure 11B). The ssGSEA results revealed that the high-risk group had higher score of immune cells such as CD8^+^ T cells and Macrophage (Figure 11C). The high-risk group had higher APC scores for co-stimulation, Check-point, inflammation promoting, parainflammation and other immune functions were higher (Figure 11D).

### Analysis of drug sensitivity

To further improve the prognosis of KIRC, we investigated the relationship between riskScore and the IC50 value of various drugs. The IC50 values for bosutinib, camptothecin, gefitinib, sunitinib and parthenolide were lower in the high-risk group, indicating that there was greater sensitivity to these drugs in high-risk patients (all *p* < 0.001, Figures 11E–J). The IC50 value of lapatinib, on the other hand, was higher in the high-risk group (Figure 11H).

### Biological Functions of *lncRNAs*

**FIGURE 12:**
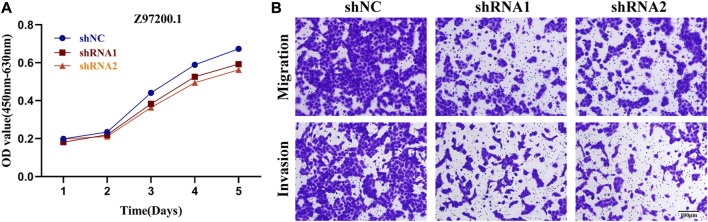
Z97200.1 stimulated the proliferation, migration and invasion of ACHN cells. **(A)** ACHN cells growth rates at 1, 2, 3, 4, and 5 days after knockdown of Z97200.1 were measured using a CCK-8 assay. **(B)** Transwell assays were conducted to assess whether Z97200.1 knockdown affected the invasion and migration of ACHN cells.

Based on the results of gene expression at the cellular level, we selected two *lncRNAs* (Z97200.1, AC093001.1) with the greatest differences in expression between normal kidney cells and KIRC cells and the most meaningful *p* values for functional experiments. CCK-8 assay revealed that the absorbance (OD) values of the Z97200.1-interfered and AC093001.1-interfered groups were significantly lower and cell proliferation was slower compared to the control group (Figure 12A and Supplementary Figure S4A). By transwell assay, it was found that the number of cells crossing the transwell chamber was significantly reduced in the Z97200.1-interfered group compared to the control group (Figure 12B). However, there was no significant change in the number of cells crossing the transwell in the AC093001.1-interfered group compared to the control group (Supplementary Figure S4B). The above experimental results indicated that high expression of Z97200.1 promoted the migration and invasion of KIRC cells.

## Discussion

The RCC was one of the most common varieties of urinary tract cancer. In clinical practice, the first-line drugs for KIRC were still tyrosine kinase inhibitors. However, due to individual heterogeneity, drug resistance occurred more often in some patients. To that end, new biomarkers must be discovered to improve the diagnosis and prognosis of KIRC. Numerous tumors were influenced by chromatin regulators to date. The high mobility group A1 (HMGA1), a chromatin regulator, had been shown to suppress BRCA1 gene expression in human breast cancer (Baldassarre et al., 2003). A study by Ding et al. identified Brahma-related gene 1 (BRG1) as a target for PTEN-deficient prostate cancer therapy (Ding et al., 2019). There was also an increasing number of studies on the relationship between prognostic models and kidney cancer. Based on a model of genes associated with ferroptosis and a model of genes associated with lactate, Hong, Sun et al. found that the OS of KIRC could be predicted using these models (Hong et al., 2021; Sun et al., 2022). Currently, *lncRNAs* had been extensively studied in RCC (Zhai et al., 2017; Yang et al., 2018; Guo et al., 2021). Tang et al. found that a model constructed from *lncRNAs* associated with ferroptosis and a model constructed from *lncRNAs* associated with pyroptosis could both be used to predict OS in KIRC (Tang et al., 2021; Tang et al., 2022). Yu et al. constructed a model consisting of *lncRNAs* associated with M6A and demonstrated that it could predict the prognosis of KIRC independently (Yu et al., 2021). However, no chromatin regulator-related *lncRNAs* had been studied in KIRC.

Differentially Expressed Chromatin Regulator-related *lncRNAs* (DECRLs) were identified using the TCGA database. Then, univariate and multivariate regression analyses were conducted to build a prognostic risk signature containing 8 *lncRNAs* (LINC00551, AL031722.1, AC093001.1, NDUFB2-AS1, LINC00894, Z97200.1, AC006160.1, and AC092422.1). We further performed survival and ROC analyses on the prognostic signature consisting of these 8 *lncRNAs*. Three datasets were used to validate the reliability of the signature. Univariate/multivariate Cox regression analysis demonstrated that the model could independently influence overall survival in KIRC. A nomogram was developed to further predict the 1-, 3- and 5-year survival rates of KIRC patients. In prognostic signature, three *lncRNAs* have been identified to be involved in tumor progression or as tumor prognostic markers. The LINC00551 was reported to reduce HSP27 phosphorylation and thus inhibit the proliferation and invasion of esophageal squamous cell carcinoma cells (Peng et al., 2021). Furthermore, LINC00551 has been shown to decrease the proliferation and invasion of esophageal squamous cell carcinoma cells by reducing HSP27 phosphorylation (Wang et al., 2020). Meng et al. demonstrated that LINC00894 expression was elevated in breast cancer cells, which promoted their proliferation and migration (Meng et al., 2021). There was only one publication on AL031722.1, involvement in the construction of a prognostic signature for low-grade gliomas (Lin et al., 2020). However, the remaining five *lncRNAs* have been little studied so far, and whether they are involved in the progression of KIRC remains to be further confirmed experimentally.

We further investigated the biological processes and signaling pathways involved in the prognostic signature constructed based on chromatin regulators by GO, KEGG and GSVA analyses. This signature was found to be involved in the IL-17 and HIF-1 signaling pathways, among others. Interleukin 17 (IL-17), a pro-inflammatory cytokine, had a crucial role in tumor formation (Nardinocchi et al., 2015; Qian et al., 2017; Zhao et al., 2020). In breast cancer, Chen et al. showed that estrogen receptors down-regulated PD-1/PD-L1 expression by regulating the IL-17 signaling pathway (Shuai et al., 2020). The HIF-1α and HIF-1β are comprised of the transcription factor hypoxia-inducible factor (HIF-1). In the study of solid tumors, the HIF-1 signaling pathway was frequently mentioned (Vaupel and Mayer, 2007; Bertout et al., 2008). The HIF-1α has been demonstrated to play an inhibitory role in KIRC (Schödel et al., 2016). The PHD3 has been proposed to cause neovascular dysplasia in pancreatic ductal adenocarcinoma through the HIF-1 signaling pathway (Tanaka et al., 2015). These pathways were involved in the biological process of numerous tumors. Based on the findings of the pathway analysis in this study, we can postulate that the model may affect KIRC through these pathways, however, this needs to be validated by further studies.

TME and immune cell infiltration have significant effects on tumor progression (Mlecnik et al., 2016; Malka et al., 2020). In this study, the prognostic signature was correlated with ESTIMATEScore and ImmuneScore. The ESTIMATEScore and ImmuneScore, on the other hand, reflected the purity of immune cells and the level of immune cell infiltration in the tumor tissue. Immune cell infiltration was shown to influence tumorigenesis and recurrence and played a critical role in immunotherapy and clinical outcomes of tumors. A higher level of macrophage infiltration was associated with the aggressiveness of human breast cancer (Acerbi et al., 2015). Hepatocellular carcinoma scoring system based on immune cell infiltration could predict patient prognosis and guide immunotherapy (Yang et al., 2021). According to Bai et al., patients with the high tumor immune infiltration group had a better prognosis and were likely to benefit more from immunotherapy (Bai et al., 2021).

The conventional view was that in most malignancies, patients with high infiltration of CD8^+^ T cells had a better prognosis. However, the impact of the degree of CD8^+^ T cell infiltration in KIRC tissue on patient prognosis remained controversial. Some studies suggested that the prognosis of KIRC patients with high CD8^+^ T cell infiltration was worse, while others had put forward the opposite view (Davis et al., 2020). Combined with the results in this paper, we found that higher risk scores suggested a worse prognosis and that high risk was associated with high infiltration of CD8^+^ T cells. In addition, some CD8^+^ T cells infiltrated in KIRC were found to express CXCL13, a chemokine. High expression of this subpopulation of CXCL13 and CD8 protein-positive T cells resulted in immune escape, leading to a worse prognosis for patients with KIRC with high infiltration of CD8^+^ T cells (Dai et al., 2021). In this paper, whether the presence of CXCL13 expression in CD8^+^ T cells was associated with poorer prognosis remains to be further verified in subsequent experiments. This study concluded that this model and immune cell infiltration were significantly correlated, suggesting that the prognostic model may influence the prognosis of KIRC by modulating tumor immune cell infiltration. However, this needed to be validated by further tests to confirm the mechanisms involved.

Immunotherapy was gaining more and more clinical and scientific attention due to its effectiveness and less side effects. Immune checkpoint inhibition therapy was one of the most important methods (Sharma and Allison, 2015). In recent years, immune checkpoint inhibitors, represented by CTLA-4 monoclonal antibody and PD-1/PD-L1 monoclonal antibody, had achieved more satisfactory results in the treatment of KIRC (Motzer et al., 2015). Currently, molecular targeting agents targeting the PD1/PD-L1 pathway, such as nivolumab, pembrolizumab and avelumab, had been successfully applied in the clinical treatment of KIRC. In this paper, PD-1 and CTLA-4 expression were higher in the high-risk group, which may explain the poor prognosis of patients in the high-risk group. In this study, we found that riskScore was correlated with immune checkpoints such as CTLA-4 and PD-1 by correlation analysis, and thus hypothesized that riskScore may influence the patient’s response to immunotherapy by modulating the immune checkpoint. In addition, we analyzed the IC50 value of some clinical drugs and found that the IC50 value were different in different risk groups. However, KIRC was not sensitive to radiotherapy, so the drug treatment for KIRC patients still needed to be discussed.

Overall, this study had certain advantages. This was the first exploration of the role of prognostic signature constructed on the basis of chromatin regulator-related *lncRNAs* in KIRC. Further, we used PCR to verify the expression of these 8 *lncRNAs* in KIRC at tissue level and cellular level. In addition, we explored the possible enrichment pathways in prognostic signature and the relationship with TME and immune response. We also selected relevant *lncRNAs* in the model and investigated its effect on the biological function of KIRC cells. However, our study also had some limitations. The prognostic signature was verified by the TCGA dataset, and the follow-up needed to be validated by other databases. Secondly, the prediction efficiency of the signature for 1- and 3-year survival of KIRC was lower than that of stage. Besides, the expression validation at tissue level and cellular level was slightly different from the expression of *lncRNAs* in the database, which may be related to the small sample size. Finally, the mechanism of prognostic models involved in regulating KIRC remained to be confirmed.

## Conclusion

In general, we constructed a prognostic signature based on 8 chromatin regulator-related *lncRNAs*, which was useful for clinicians to determine the prognosis of KIRC. Furthermore, the signature exhibited tremendous potential in evaluating TME and immunotherapy in KIRC patients. More studies are needed to validate this signature in the future.

## Data Availability

The original contributions presented in the study are included in the article/Supplementary Material, further inquiries can be directed to the corresponding authors.

## Supplementary material

SUPPLEMENTARY FIGURE S1 Verification of prognostic significance of these 8 CRlncRNAs.

SUPPLEMENTARY FIGURE S2 The correlation between these 8 CRlncRNAs and immune cells.

SUPPLEMENTARY FIGURE S3
**(A)** Difference between high and low risk group. **(B)** Kaplan–Meier curve of different groups.

SUPPLEMENTARY FIGURE S4 Biological Functions of AC093001.1 in 769-P cells **(A)**. CCK-8 assay was conducted to evaluate whether AC093001.1 knockdown affected the proliferation of 769-P cells. **(B)** the invasion and migration of different groups of 769-P cells.
