# Supplementary figures and images for: Chromatin regulators-related lncRNA signature predicting the prognosis of kidney renal clear cell carcinoma and its relationship with immune microenvironment: A study based on bioinformatics and experimental validation
Source: Front Genet. 2022 Oct 20;13:974726. doi: 10.3389/fgene.2022.974726 (PMC9630733; doi:10.3389/fgene.2022.974726)

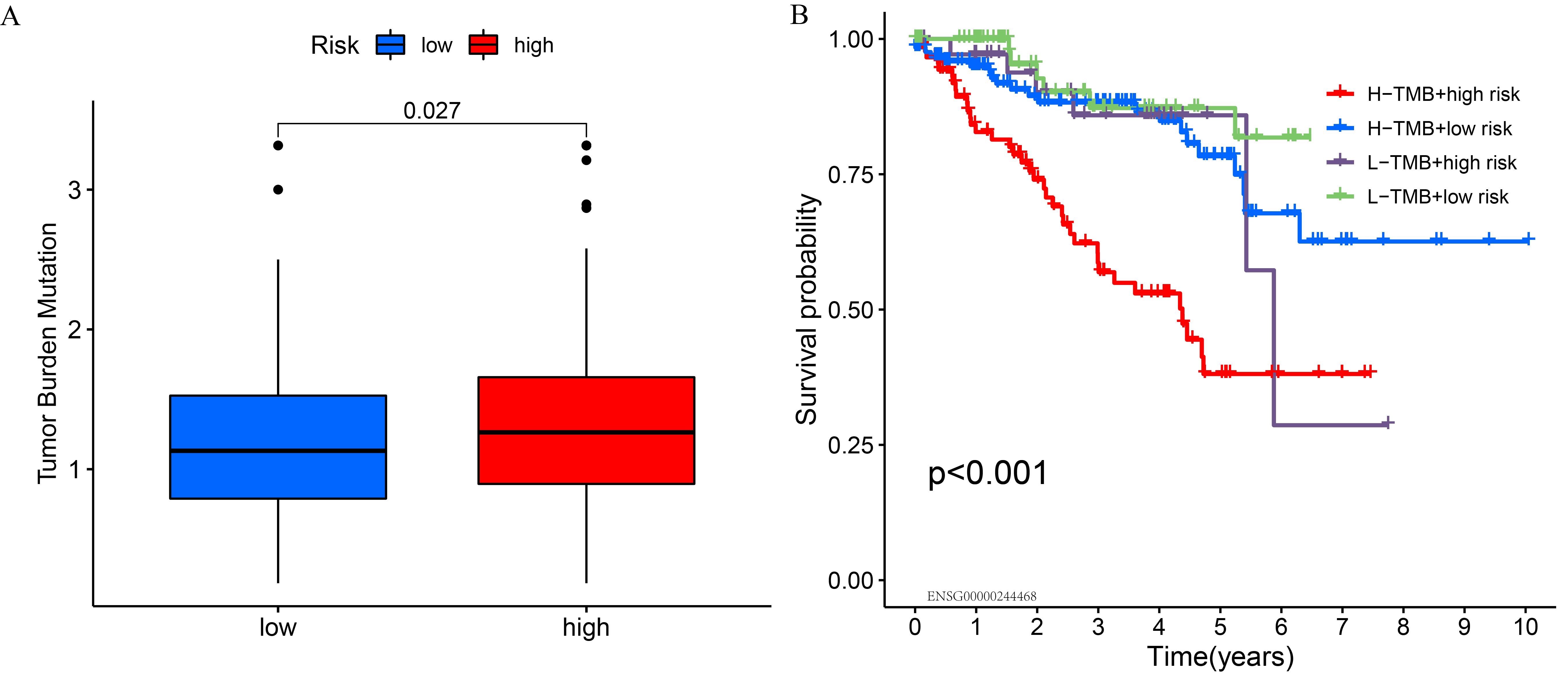

Supplement: Supplementary file 1 [file Image3.tif]

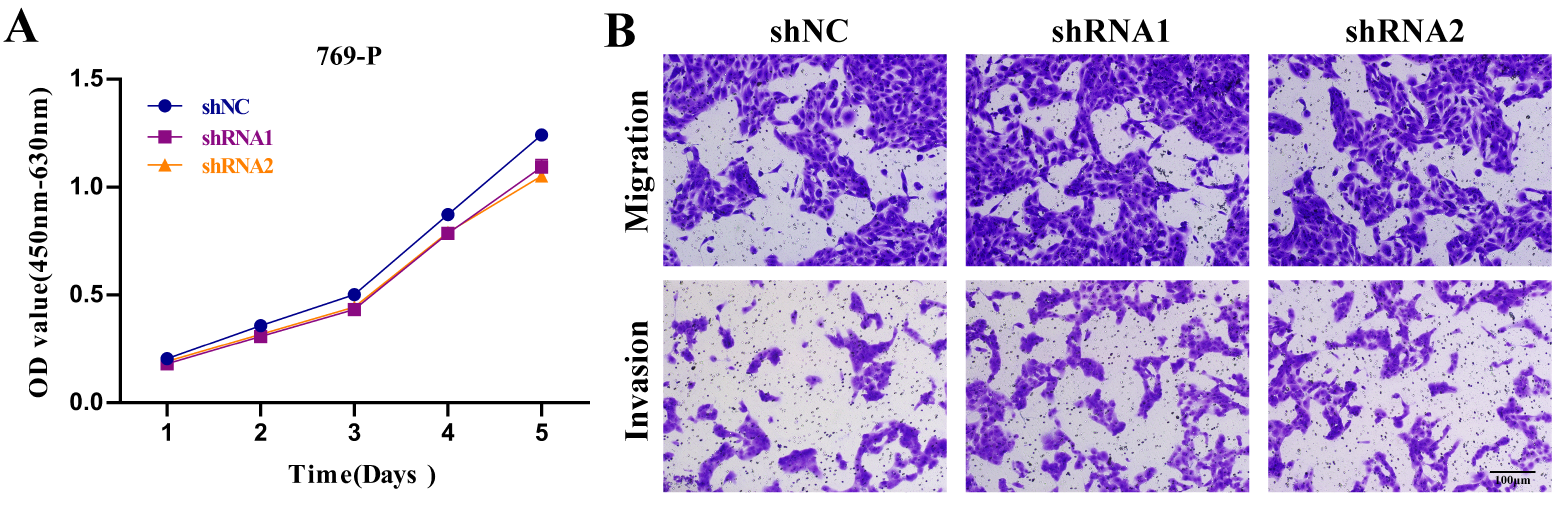

Supplement: Supplementary file 2 [file Image4.tif]

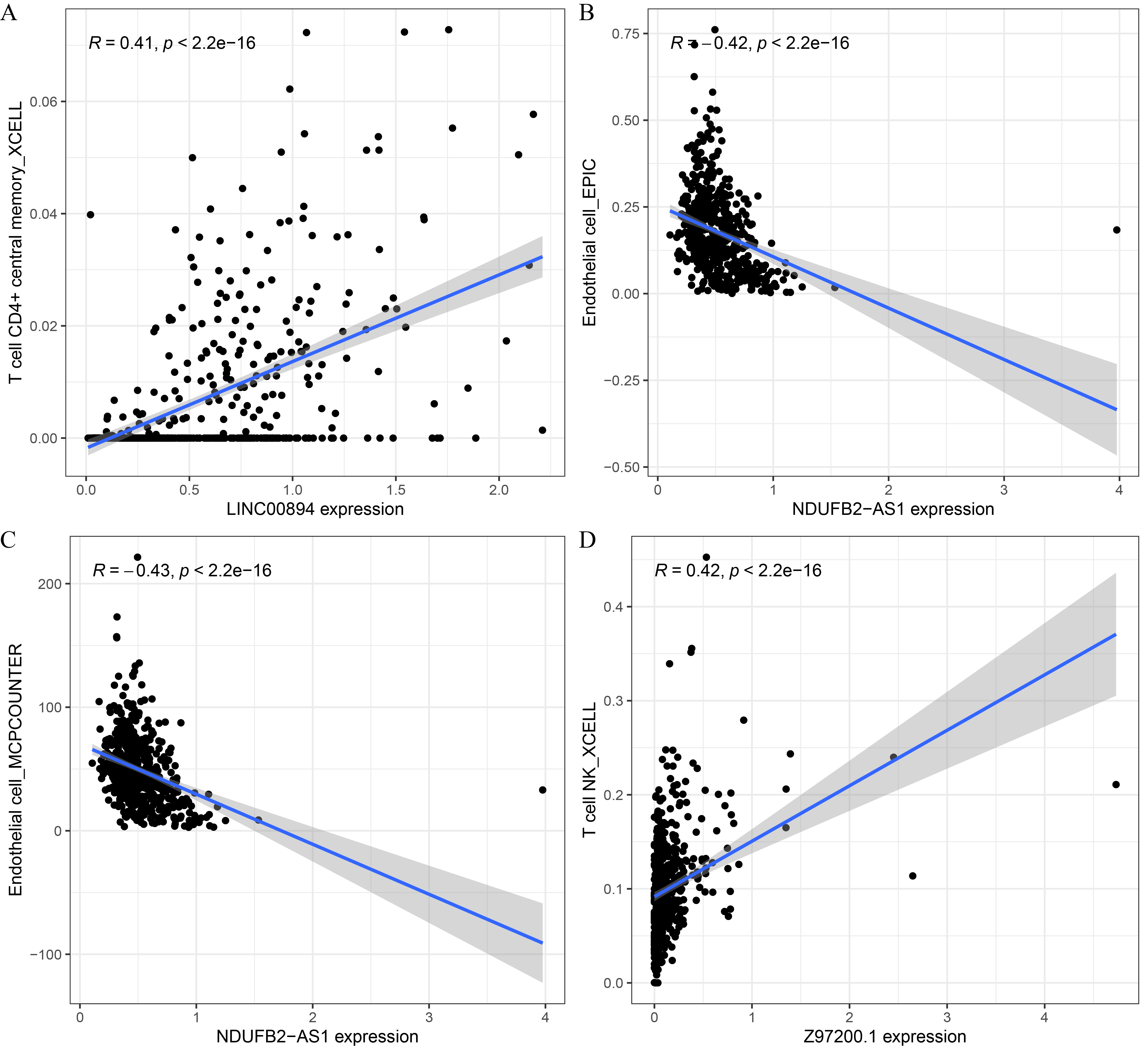

Supplement: Supplementary file 3 [file Image2.tif]

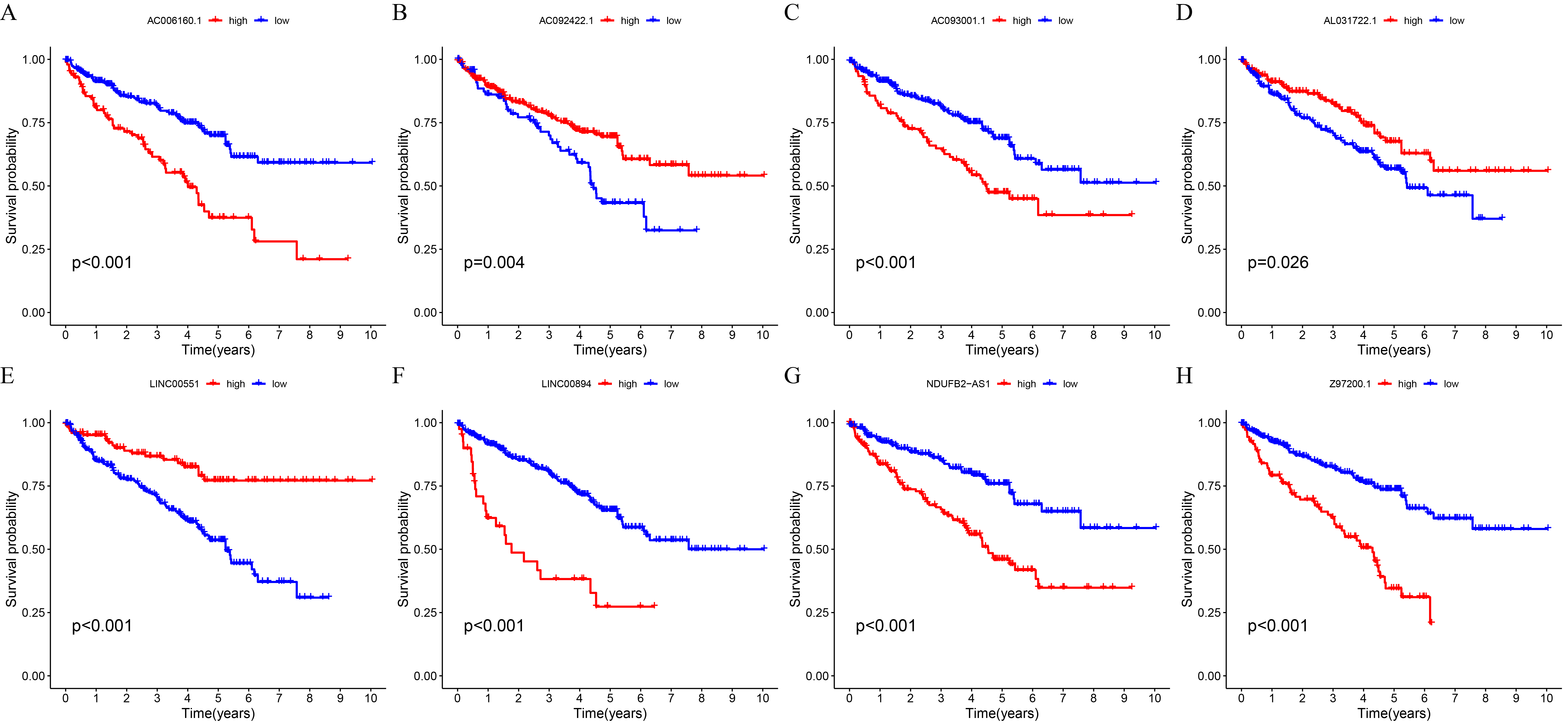

Supplement: Supplementary file 4 [file Image1.tif]
