# Supplementary material for: Chromatin regulators-related lncRNA signature predicting the prognosis of kidney renal clear cell carcinoma and its relationship with immune microenvironment: A study based on bioinformatics and experimental validation
Source: Front Genet. 2022 Oct 20;13:974726. doi: 10.3389/fgene.2022.974726 (PMC9630733; doi:10.3389/fgene.2022.974726)
Supplement: Supplementary file 5 [file Table1.doc]

**Supplemental Table 1 Univariate cox regression analysis of Differentially expressed CRrlncRNAs**

| **ID** | **HR** | **HR.95L** | **HR.95H** | **pvalue** |
| --- | --- | --- | --- | --- |
| ZNF32-AS2 | 1.6392 | 1.1185 | 2.4021 | 0.0113 |
| AC023509.3 | 2.0084 | 1.4166 | 2.8476 | <0.001 |
| GK-IT1 | 1.3753 | 1.0527 | 1.7968 | 0.0195 |
| ANKRD10-IT1 | 1.3363 | 1.0807 | 1.6522 | 0.0074 |
| C8orf44 | 2.1309 | 1.4437 | 3.1450 | 0.0001 |
| AC005363.2 | 1.5530 | 1.0117 | 2.3841 | 0.0441 |
| AP001001.1 | 2.9323 | 1.4286 | 6.0186 | 0.0034 |
| LINC00551 | 0.0466 | 0.0080 | 0.2719 | 0.0007 |
| AL031722.1 | 0.6889 | 0.4935 | 0.9617 | 0.0286 |
| AC025176.1 | 1.5036 | 1.0901 | 2.0738 | 0.0129 |
| AC008764.6 | 1.6060 | 1.1880 | 2.1709 | 0.0021 |
| SKAP1-AS1 | 1.4138 | 1.0020 | 1.9949 | 0.0487 |
| ACBD3-AS1 | 2.6208 | 1.5462 | 4.4423 | 0.0003 |
| AC010531.6 | 1.2900 | 1.0164 | 1.6372 | 0.0363 |
| AC005519.1 | 1.5603 | 1.0737 | 2.2675 | 0.0197 |
| AL135999.1 | 1.7271 | 1.2662 | 2.3556 | 0.0006 |
| AC008735.2 | 1.3193 | 1.0983 | 1.5847 | 0.0031 |
| AC005332.2 | 3.0428 | 1.0320 | 8.9713 | 0.0437 |
| AC243960.1 | 1.6107 | 1.1745 | 2.2090 | 0.0031 |
| AC090589.3 | 1.5499 | 1.1276 | 2.1303 | 0.0069 |
| AC012615.6 | 1.7333 | 1.2075 | 2.4880 | 0.0029 |
| CD44-AS1 | 4.5608 | 2.7292 | 7.6217 | <0.001 |
| AC244093.5 | 3.3372 | 1.3148 | 8.4705 | 0.0112 |
| AC004923.4 | 1.4108 | 1.1036 | 1.8036 | 0.0060 |
| CR936218.1 | 1.4956 | 1.0730 | 2.0846 | 0.0175 |
| AP000873.2 | 1.8719 | 1.0803 | 3.2438 | 0.0254 |
| AC021078.1 | 1.4791 | 1.1342 | 1.9289 | 0.0039 |
| AL135818.1 | 1.9160 | 1.0793 | 3.4015 | 0.0264 |
| AL355075.2 | 1.5681 | 1.0765 | 2.2841 | 0.0191 |
| AC027601.1 | 3.6409 | 1.8865 | 7.0271 | 0.0001 |
| AL161668.3 | 1.5276 | 1.0630 | 2.1951 | 0.0220 |
| AC093001.1 | 1.2293 | 1.0602 | 1.4254 | 0.0063 |
| AC002128.1 | 1.7097 | 1.1634 | 2.5125 | 0.0063 |
| AC008764.8 | 2.2815 | 1.4529 | 3.5826 | 0.0003 |
| ARHGAP27P1-BPTFP1-KPNA2P3 | 1.4524 | 1.0957 | 1.9252 | 0.0094 |
| AC008870.2 | 2.7468 | 1.7918 | 4.2108 | <0.001 |
| NDUFB2-AS1 | 1.6046 | 1.1257 | 2.2872 | 0.0089 |
| AC066613.1 | 1.6408 | 1.0017 | 2.6876 | 0.0492 |
| AC010618.3 | 1.4396 | 1.0502 | 1.9733 | 0.0236 |
| LINC00342 | 1.4457 | 1.1575 | 1.8058 | 0.0012 |
| AC005104.1 | 1.7088 | 1.2728 | 2.2942 | 0.0004 |
| AC040162.3 | 2.6078 | 1.5844 | 4.2922 | 0.0002 |
| AC010422.2 | 1.5100 | 1.0554 | 2.1605 | 0.0242 |
| AD001527.1 | 1.3517 | 1.0725 | 1.7035 | 0.0107 |
| AC092301.1 | 2.9103 | 1.3464 | 6.2904 | 0.0066 |
| AC114730.3 | 1.8909 | 1.2515 | 2.8570 | 0.0025 |
| LINC00426 | 1.7280 | 1.0990 | 2.7170 | 0.0178 |
| AC091057.1 | 4.6662 | 2.3421 | 9.2966 | <0.001 |
| AC025048.4 | 1.4120 | 1.0323 | 1.9312 | 0.0308 |
| AL391684.1 | 1.7730 | 1.1294 | 2.7833 | 0.0128 |
| MCM3AP-AS1 | 1.9313 | 1.0524 | 3.5439 | 0.0336 |
| AC073487.1 | 2.0291 | 1.3489 | 3.0525 | 0.0007 |
| AC005253.1 | 1.6297 | 1.0993 | 2.4162 | 0.0151 |
| BMS1P4 | 7.3039 | 2.1914 | 24.3435 | 0.0012 |
| AC087289.2 | 3.7082 | 1.9182 | 7.1685 | <0.001 |
| AC079907.1 | 1.5146 | 1.0988 | 2.0877 | 0.0112 |
| PTOV1-AS2 | 1.3918 | 1.1329 | 1.7098 | 0.0016 |
| AC117490.2 | 1.5868 | 1.0547 | 2.3874 | 0.0267 |
| AC009120.2 | 1.4738 | 1.1175 | 1.9437 | 0.0060 |
| AL049552.1 | 1.5061 | 1.0169 | 2.2306 | 0.0410 |
| AL031670.1 | 1.9026 | 1.2627 | 2.8667 | 0.0021 |
| Z97832.2 | 2.3328 | 1.2672 | 4.2946 | 0.0065 |
| AC004585.1 | 1.3595 | 1.0379 | 1.7807 | 0.0258 |
| N4BP2L2-IT2 | 1.9746 | 1.3369 | 2.9165 | 0.0006 |
| LIF-AS1 | 1.4223 | 1.0154 | 1.9923 | 0.0405 |
| AC002553.2 | 2.1124 | 1.4924 | 2.9900 | <0.001 |
| LINC00894 | 2.4833 | 1.6359 | 3.7697 | <0.001 |
| AC002553.1 | 1.6623 | 1.2081 | 2.2872 | 0.0018 |
| AP002907.1 | 2.2039 | 1.4292 | 3.3986 | 0.0003 |
| AC079203.1 | 1.5983 | 1.2054 | 2.1193 | 0.0011 |
| RUSC1-AS1 | 1.5192 | 1.1245 | 2.0523 | 0.0064 |
| CCDC18-AS1 | 1.6050 | 1.2462 | 2.0672 | 0.0002 |
| AC015849.1 | 1.7230 | 1.0875 | 2.7298 | 0.0205 |
| AC010618.2 | 1.4683 | 1.1063 | 1.9487 | 0.0078 |
| HMGA1P4 | 1.3239 | 1.0649 | 1.6460 | 0.0115 |
| FAM13A-AS1 | 1.4697 | 1.0846 | 1.9916 | 0.0130 |
| RNF139-AS1 | 4.2410 | 2.0354 | 8.8367 | 0.0001 |
| AC130469.1 | 1.3517 | 1.0799 | 1.6919 | 0.0085 |
| AP002490.1 | 1.9187 | 1.0075 | 3.6540 | 0.0474 |
| AC127024.5 | 1.6351 | 1.1854 | 2.2553 | 0.0027 |
| AC138207.4 | 1.5795 | 1.0864 | 2.2966 | 0.0167 |
| AC129510.1 | 1.7564 | 1.3029 | 2.3677 | 0.0002 |
| Z97200.1 | 1.4526 | 1.1190 | 1.8856 | 0.0050 |
| AC006160.1 | 1.3873 | 1.0147 | 1.8967 | 0.0402 |
| FMR1-IT1 | 1.5464 | 1.0551 | 2.2665 | 0.0254 |
| PSPC1-AS2 | 2.2039 | 1.4921 | 3.2553 | <0.001 |
| AC092422.1 | 0.0962 | 0.0141 | 0.6576 | 0.0170 |
| AC084824.3 | 2.2439 | 1.2616 | 3.9910 | 0.0059 |
| ZKSCAN2-DT | 1.9457 | 1.3701 | 2.7630 | 0.0002 |
| AL513327.1 | 1.6244 | 1.1583 | 2.2780 | 0.0049 |
| MHENCR | 1.4576 | 1.1906 | 1.7845 | 0.0003 |
| AC099850.3 | 1.3944 | 1.0831 | 1.7953 | 0.0099 |
| AC005306.1 | 2.2706 | 1.4262 | 3.6151 | 0.0005 |
| ALG13-AS1 | 1.4274 | 1.0104 | 2.0163 | 0.0435 |
| NARF-IT1 | 2.3046 | 1.4151 | 3.7532 | 0.0008 |
| AC127024.4 | 1.4403 | 1.1042 | 1.8788 | 0.0071 |
| YEATS2-AS1 | 2.5954 | 1.5926 | 4.2296 | 0.0001 |
| PCED1B-AS1 | 1.4328 | 1.1149 | 1.8413 | 0.0050 |
| AC020907.4 | 1.7392 | 1.3346 | 2.2663 | <0.001 |
