# Supplementary material for: Chromatin regulators-related lncRNA signature predicting the prognosis of kidney renal clear cell carcinoma and its relationship with immune microenvironment: A study based on bioinformatics and experimental validation
Source: Front Genet. 2022 Oct 20;13:974726. doi: 10.3389/fgene.2022.974726 (PMC9630733; doi:10.3389/fgene.2022.974726)
Supplement: Supplementary file 7 [file Table2.doc]

| The training set | | | | | | | | |
| --- | --- | --- | --- | --- | --- | --- | --- | --- |
| Univariate cox regression analysis | | | | | Multivariate cox regression analysis | | | |
| ID | HR | HR.95L | HR.95H | p value | HR | HR.95L | HR.95H | p value |
| Age | 1.028629 | 1.011938 | 1.045596 | 0.000720 | 1.032072 | 1.013502 | 1.050982 | 0.000655 |
| Gender | 1.003782 | 0.676791 | 1.488758 | 0.985024 | 1.144356 | 0.758930 | 1.725522 | 0.519888 |
| Grade | 2.468466 | 1.906298 | 3.196416 | 7.24E-12 | 1.426038 | 1.062846 | 1.913340 | 0.017964 |
| Stage | 2.017223 | 1.709594 | 2.380209 | 9.41E-17 | 1.720056 | 1.419514 | 2.084229 | 3.11E-08 |
| riskScore | 1.257917 | 1.185914 | 1.334292 | 2.35E-14 | 1.165713 | 1.087941 | 1.249043 | 1.35E-05 |
| The testing set | | | | | | | | |
| Univariate cox regression analysis | | | | | Multivariate cox regression analysis | | | |
| ID | HR | HR.95L | HR.95H | p value | HR | HR.95L | HR.95H | p value |
| Age | 1.037579 | 1.013863 | 1.061850 | 0.001766 | 1.042135 | 1.015158 | 1.069828 | 0.002041 |
| Gender | 0.895514 | 0.516166 | 1.553657 | 0.694632 | 1.045091 | 0.593910 | 1.839025 | 0.878431 |
| Grade | 2.142835 | 1.462873 | 3.138853 | 9.11E-05 | 1.588245 | 1.020690 | 2.471389 | 0.040292 |
| Stage | 1.681434 | 1.333529 | 2.120103 | 1.12E-05 | 1.404069 | 1.055865 | 1.867104 | 0.019607 |
| riskScore | 1.378750 | 1.181764 | 1.608572 | 4.44E-05 | 1.257515 | 1.061753 | 1.489371 | 0.007954 |
| The entire set | | | | | | | | |
| Univariate cox regression analysis | | | | | Multivariate cox regression analysis | | | |
| ID | HR | HR.95L | HR.95H | p value | HR | HR.95L | HR.95H | p value |
| Age | 1.031951 | 1.018340 | 1.045744 | 3.44E-06 | 1.036203 | 1.020916 | 1.051719 | 2.74E-06 |
| Gender | 0.954280 | 0.694207 | 1.311784 | 0.773142 | 1.070399 | 0.770870 | 1.486313 | 0.684604 |
| Grade | 2.319748 | 1.878620 | 2.864458 | 5.32E-15 | 1.444864 | 1.139701 | 1.831737 | 0.002364 |
| Stage | 1.904997 | 1.665917 | 2.178389 | 4.55E-21 | 1.626228 | 1.391688 | 1.900296 | 9.40E-10 |
| riskScore | 1.268425 | 1.202999 | 1.337410 | 1.37E-18 | 1.177102 | 1.106929 | 1.251724 | 2.00E-07 |

**Table 1 Univariate and multivariate Cox regression analysis of TCGA dataset**
